# Inhibitory effect of Bofutsushosan (Fangfengtongshengsan) extract on the absorption of fructose in rats and mice

**DOI:** 10.1007/s11418-023-01697-8

**Published:** 2023-04-11

**Authors:** Kohei Takagi, Takashi Sugihira, Miho Kitamura, Mami Kawai, Yoko Mitsuguchi, Kosei Tsukamoto, Hirofumi Nakanishi, Toshiaki Makino

**Affiliations:** 1 Basic Research and Development Division, Rohto Pharmaceutical Co., Ltd., 6-5-4 Kunimidai, Kizugawa, Kyoto 619-0216 Japan; 2 Safety Design Center, Rohto Pharmaceutical Co., Ltd., 6-5-4 Kunimidai, Kizugawa, Kyoto 619-0216 Japan; 3 Internal Medicine and Functional Food Development Division, Rohto Pharmaceutical Co., Ltd., 6-5-4 Kunimidai, Kizugawa, Kyoto 619-0216 Japan; 4 Department of Pharmacognosy, Graduate School of Pharmaceutical Sciences, Nagoya City University, 3-1 Tanabe-dori, Mizuho-ku, Nagoya 467-8603 Japan

**Keywords:** Bofutsushosan, Fructose, *Rheum palmatum* rhizome, Rhubarb, Sennoside A

## Abstract

Bofutsushosan (BTS; fangfengtongshengsan in Chinese) is a formula in traditional Japanese Kampo and Chinese medicine comprising 18 crude drugs and used to treat obesity and metabolic syndrome. In our previous study, BTS boiling water extract inhibited the uptake of fructose absorbed via glucose transporter 5 into cultured cells. In this study, the inhibitory effect of BTS extract on the absorption of fructose from the intestine was investigated in vivo. The extract of BTS was orally administered to rats at doses equivalent to 25-fold of the daily dose for humans. One minute after sample administration, fructose was orally administered and blood samples were collected from the jugular vein 0.5, 1, 1.5, 2, and 4 h after the administration of fructose. The absorption of fructose from the intestine was significantly reduced by treatment with BTS extract, and this in vivo study reproduced previous in vitro results. Subsequently, the blood samples were collected from the portal vein 30 min after the oral administration of fructose in mice. BTS extract significantly reduced fructose absorption in mice, and compared the effect of modified BTS samples by removing one to several crude drugs from BTS. We found that the dried rhizome of *Rheum palmatum* (RR) significantly contributed to the inhibitory effect of BTS on fructose absorption. We found sennoside A to be the active ingredient of RR for the inhibition of fructose absorption, and that its effect almost saturated at a dose of 3 mg/kg. These results support the action mechanisms of BTS when used for the treatment of obesity in clinics and drug stores.

**Graphical abstract:**

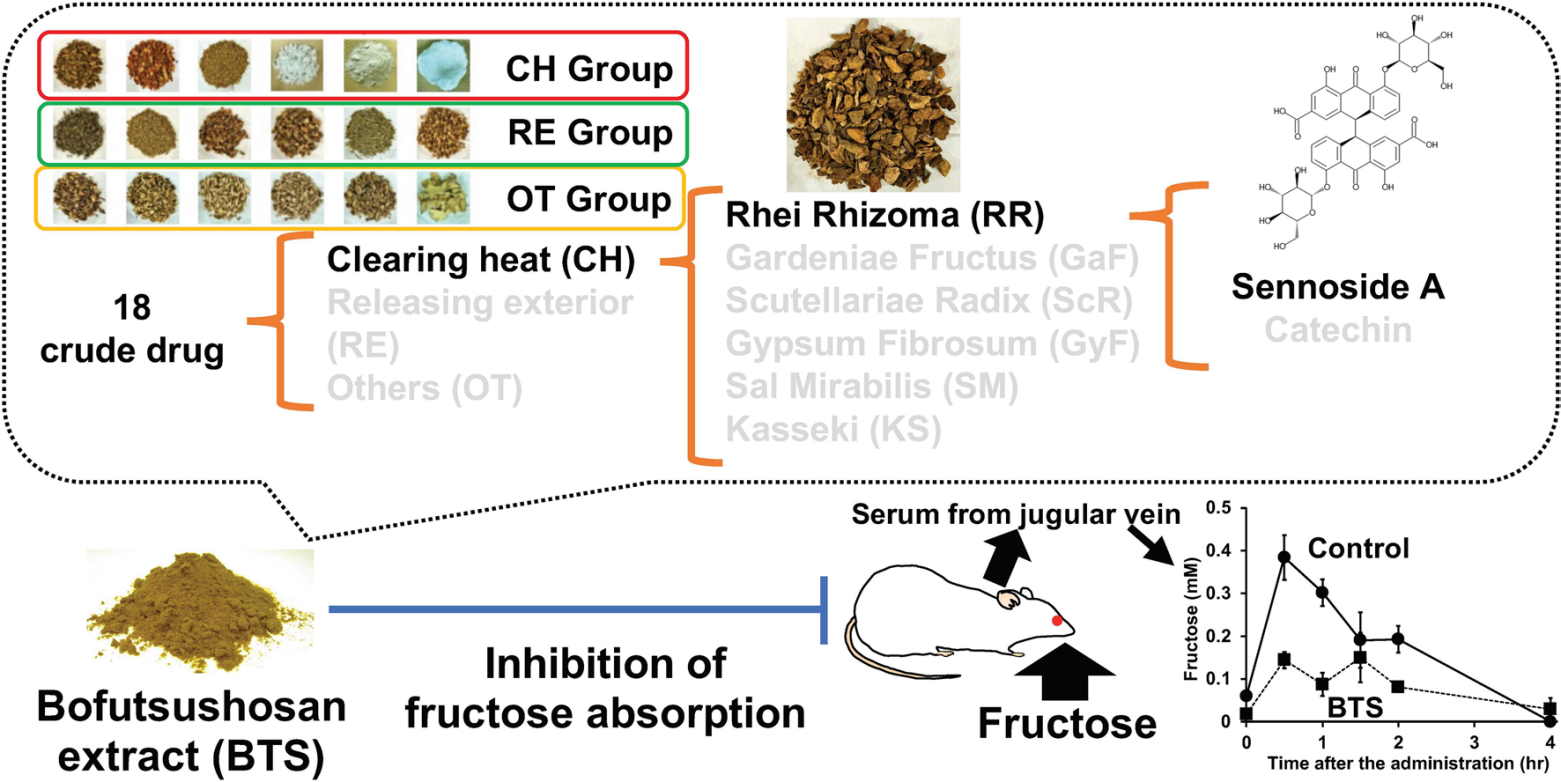

## Introduction

The National Health and Nutrition Survey in Japan in 2019 revealed that 33.0% of men and 22.3% of women with 20-years old or higher were obese (body mass index > 25.0), that these values have not been changed in women but have increased in men in the past 10 years, and that these values in men were higher in older than younger generations; 23.1% of men in their 20 s, 39.7% of those in their 40 s, and 39.2% of those in their 50 s [1]. The Japan Society for the Study of Obesity defines obesity as a condition requiring medical weight reduction associated with or expected to be associated with obesity-related health disorders, particularly in cases of visceral fat accumulation [2].

Excessive intake of sugar and fat as energy sources is the strong factor contributing to obesity. Among sugars, fructose has been reported to be more closely associated with metabolic diseases [3], and some experimental studies have shown that high fructose intake is associated with the development of metabolic syndrome, which is characterized by insulin resistance, hypertension, hyperlipidemia, visceral fat accumulation, and obesity, and increases the risk of cardiovascular disease [4]. When overweight or obese subjects consumed 25% of the daily energy requirement from glucose or fructose for 2 weeks in addition to their normal diet, a significant increase in visceral fat was observed in the fructose-intake group than in the glucose-treated group [5]. When children with obesity and metabolic syndrome were restricted to fructose intake, a significant reduction in visceral fat is observed [6].

Bofutsushosan (BTS; *fangfengtongshengsan* in Chinese) consists of 18 crude drugs and is used in Japanese traditional Kampo and traditional Chinese medicines to treat flabby belly, constipation, decrease in urinary volume, hyperchlorhydria, kidney diseases, heart weakening, arteriosclerosis, hypertension, cerebral hemorrhage, palpitation, shoulder discomfort, rush of blood to the head, obesity, edema, chronic nephritis, and eczema [7]. It has been shown that BTS extract has a preventive effect against hyperglycemia and body fat accumulation caused by fructose loading in in vivo study [8]. In our previous study, BTS boiling water extract inhibited the uptake of fructose absorbed via glucose transporter 5 (GLUT5) in cultured cells [9].

In this study, we investigated the inhibitory effect of BTS extract on the absorption of fructose from the intestine in rats and mice and explored the active crude drugs and ingredients in BTS.

## Materials and methods

### Preparation of extracts of BTS and each herbal component of BTS

All crude drugs used in this study were purchased from Tianjin Rohto Herbal Medicine (Tianjin, China) and were the grade of Japanese Pharmacopoeia 18th Edition (JPXVIII) [10]. The components of BTS, the origins of these crude drugs, and their daily doses in humans are shown in Table 1. We prepared the groups containing six crude drugs from the components of BTS as shown in Table 1. Furthermore, we prepared the groups containing one to several crude drugs from the components of the group CH (Table 1).

**Table 1:** The origins, distributers, and lot numbers of the crude drugs used

Group or Latin names of crude drug	Origins	Lot number^a)^
Drugs for clearing heat group (CH)		
Rhei Rhizoma (RR)	The dried rhizome of *Rheum palmatum* Linné	40807171, 9931162, TA272624
Gardeniae Fructus (GaF)	The dried fruit of *Gardenia jasminoides* J. Ellis	S322525, TA463105
Scutellariae Radix (ScR)	The dried root of *Scutellaria baicalensis* Georgi	TD192901, TH412425
Gypsum Fibrosum (GyF)	Natural hydrous calcium sulfate	UA462623
Sal Mirabilis (SM)	A mineral substance, mainly composed of sodium sulfate hydrate	ZA462123
Kasseki (KS)	A mineral substance, mainly composed of aluminium silicate hydrate and silicon dioxide	S322524, S272211
Drugs for releasing exterior group (RE)		
Ephedrae Herba (EH)	The dried terrestrial stem of *Ephedra sinica* Stapf	UA362322, UB363015
Forsythiae Fructus (FF)	The dried fruit of *Forsythia suspensa* (Thunberg) Vahl	TA272624, UA272127
Cnidii Rhizoma (CR)	The dried rhizome of *Cnidium officinale* Makino	S322525, BE412610
Menthae Herba (MH)	The dried terrestrial part of *Mentha canadensis* Linné	S322525, TH462121
Saposhnikoviae Radix (SaR)	The dried root and rhizome of *Saposhnikovia divaricata* Schischkin	9B50518
Schizonepetae Spica (SS)	The dried spike of *Nepeta tenuifolia* Bentham	TA272624, UA272127
Others (OT)		
Angelicae Radix (AnR)	The dried root of *Angelica acutiloba* (Siebold & Zucc.) Kitag	TI462612
Atractylodis Rhizoma (AtR)	The dried rhizome of *Atractylodes japonica* Koidzumi ex Kitamura	TA412207
Glycyrrhizae Radix (GR)	The dried root and stolon of *Glycyrrhiza uralensis* Fisher	TH362202
Paeoniae Radix (PaR)	The dried root of *Paeonia lactiflora* Pallas	S462518
Platycodi Radix (PlR)	The dried root of *Platycodon grandiflorum* (Jacques) A.De Candolle	UA272127
Zingiberis Rhizoma (ZR)	The dried rhizome of *Zingiber officinale* Roscoe	9AN0227

Bofutsushosan (BTS) is the mixture of 18 crude drugs shown in this Table. Among BTS, we prepared 3 sub-groups CH, RE, and OT which contains 6 crude drugs. All crude drug samples met the grade standards of the Japanese Pharmacopoeia 18th Edition (Pharmaceutical and Medical Device Regulatory Science Society of Japan, 2021). The quality managers of each distributer identified and certificated the plant species, and the voucher specimens are deposited in Rohto Pharmaceutical Co., Ltd., Japan

^a^All crude drugs were purchased from Tianjin Rohto Herbal Medicine (Tianjin, China)

The mixture of crude drugs of the daily dose of BTS or its components were boiled with 20 times the weight of H_2_O for 40 min, and filtered. Then, the decoctions were lyophilized to yield powdered extracts. The ratios of extract yielded were shown in Table 2. The powdered extracts were stored at  – 80 °C until use.

**Table 2:** The name of crude drug mixture, their doses in the group, and their ratios of extract yielded

Name of crude drug or its groups	CH^a)^ group						RE group						OT group						Ratio^b)^
	RR	GaF	ScR	GyF	SM	KS	EH	FF	R	MH	aR	SS	AnR	AtR	GR	PaR	PIR	ZR	
BTS	1.5^c)^	1.2	2.0	2.0	1.5	3.0	1.2	1.2	1.2	1.2	1.2	1.2	1.2	2.0	2.0	1.2	2.0	0.4	28
CH + OT	1.5	1.2	2.0	2.0	1.5	3.0	–	–	–	–	–	–	1.2	2.0	2.0	1.2	2.0	0.4	35
RE + OT	–	–	–	–	–	–	1.2	1.2	1.2	1.2	1.2	1.2	1.2	2.0	2.0	1.2	2.0	0.4	30
RE + CH	1.5	1.2	2.0	2.0	1.5	3.0	1.2	1.2	1.2	1.2	1.2	1.2	–	–	–	–	–	–	31
RR + GaF + ScR + RE + OT (BTS–GyF–SM–KS)	1.5	1.2	2.0	–	–	–	1.2	1.2	1.2	1.2	1.2	1.2	1.2	2.0	2.0	1.2	2.0	0.4	31
RR + ScR + GyF + SM + KS + RE + OT (BTS – GaF)	1.5	–	2.0	2.0	1.5	3.0	1.2	1.2	1.2	1.2	1.2	1.2	1.2	2.0	2.0	1.2	2.0	0.4	30
GaF + ScR + GyF + SM + KS + RE + OT (BTS – RR)	–	1.2	2.0	2.0	1.5	3.0	1.2	1.2	1.2	1.2	1.2	1.2	1.2	2.0	2.0	1.2	2.0	0.4	28
RR + GaF + GyF + SM + KS + RE + OT (BTS – ScR)	1.5	1.2	–	2.0	1.5	3.0	1.2	1.2	1.2	1.2	1.2	1.2	1.2	2.0	2.0	1.2	2.0	0.4	27
GyF + SM + KS + RE + OT (BTS–RR–GaF–ScR)	–	–	–	2.0	1.5	3.0	1.2	1.2	1.2	1.2	1.2	1.2	1.2	2.0	2.0	1.2	2.0	0.4	22
RR + GyF + SM + KS + RE + OT (BTS–GaF–ScR)	1.5	–	–	2.0	1.5	3.0	1.2	1.2	1.2	1.2	1.2	1.2	1.2	2.0	2.0	1.2	2.0	0.4	23
GaF + GyF + SM + KS + RE + OT (BTS–RR–ScR)	–	1.2	–	2.0	1.5	3.0	1.2	1.2	1.2	1.2	1.2	1.2	1.2	2.0	2.0	1.2	2.0	0.4	24
ScR + GyF + SM + KS + RE + OT (BTS–RR–GaF)	–	–	2.0	2.0	1.5	3.0	1.2	1.2	1.2	1.2	1.2	1.2	1.2	2.0	2.0	1.2	2.0	0.4	24
CH	1.5	1.2	2.0	2.0	1.5	3.0	–	–	–	–	–	–	–	–	–	–	–	–	23
RR + GaF + ScR	1.5	1.2	2.0	–	–	–	–	–	–	–	–	–	–	–	–	–	–	–	31
RR + GaF	1.5	1.2	–	–	–	–	–	–	–	–	–	–	–	–	–	–	–	–	22
RR + ScR	1.5	–	2.0	–	–	–	–	–	–	–	–	–	–	–	–	–	–	–	30
RR	1.5	–	–	–	–	–	–	–	–	–	–	–	–	–	–	–	–	–	27

^a^The abbreviations in this Table are defined in Table 1

^b^The ratio of extract yielded (%)

^c^Dairy dose of each crude drug in the groups or RR (g)

The fingerprint pattern of the BTS extract is shown in Supplemental Fig. 1. BTS extract (50 mg) was vortexed in 1 ml MeOH, and centrifuged at 1.4 × 10^4^ *g* for 7 min. The supernatant (30 µl) was injected into HPLC under the following conditions: system, Shimadzu LC–10A_*VP*_ (Kyoto, Japan); column, TSK-GEL ODS-80_TS_ (4.6 × 250 mm, Tosoh, Tokyo); mobile phase, 0.05 M AcOH – AcONH_4_ buffer (pH 3.6)/CH_3_CN 90:10 (0 min) – 0:100 (60 min), linear gradient; flow rate, 1.0 ml/min; column temperature, 40 °C; and detection, 200–400 nm by a photodiode array detector. Some peaks were identified based on the retention times and UV spectra of standard compounds.

**Fig. 1:**
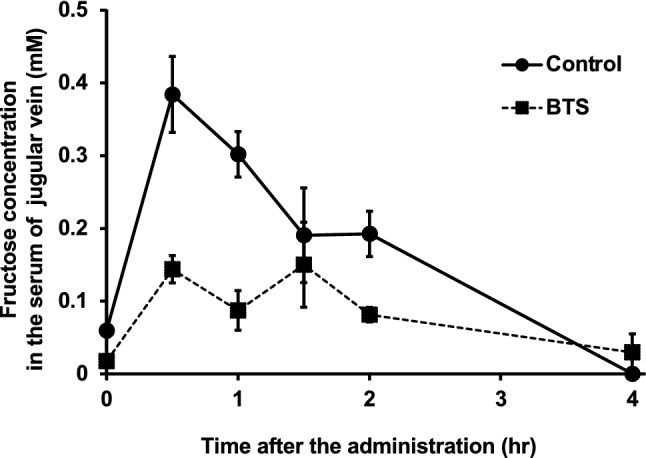
Effect of the extracts of on the absorption of fructose in rat. The extracts of Bofutsushosan (BTS; a mixture of CH + RE + OT) were prepared, and the samples were orally administered to rats at doses equivalent to 25-fold of the daily dose for humans (2.5 g/kg). One minute after sample administration, fructose (2.0 g/kg) was orally administered. At 0, 30, 60, 120, and 240 min after the administration of fructose, transvenous blood samples were collected. Data represent mean ± S.E. (*n* = 3–4). Two-way ANOVA indicated a significant main effect of BTS treatment (F_1,33_ = 25.4, *p* < 0.001), time (F_5,33_ = 14.8, *p* < 0.001), and interaction between BTS treatment and time (F_5,33_ = 4.58, *p* < 0.001)

### Animal experiments

All animal care and experimental procedures were performed in accordance with the laws and guidelines of the Japanese government and approved by the Animal Care Committee of Rohto Pharmaceutical Co.

Specific pathogen-free (SPF) male Wistar rats (8 week-old; Charles River Laboratories Japan, Yokohama, Japan) and C57BL/6 J mice (7 week-old; Clea Japan, Tokyo, Japan) were housed at 21–25 °C under a 12 h light–dark cycle and fed with a CE-2 diet (γ-ray-irradiated; Clea) and had water available ad libitum. Before oral administration, animals were acclimated to their housing for a week.

The extracts of BTS, mixtures of crude drugs, sennoside A (Fujifilm Wako Pure Chemical Industries, Osaka), and ( +)-catechin hydrate (Sigma-Aldrich, St. Louis, MO, USA) were dissolved in H_2_O at doses equivalent to BTS corresponding to 25-fold of the daily dose for humans, as shown in Table 2, and were orally administered to animals. In the previous study using BTS extract, the doses of BTS were 20- and 40-fold of the daily dose for humans, and the 40-fold dose of BTS inhibited the absorptions of both lipids and cholesterol but the 20-fold dose of BTS inhibited that of only cholesterol in mice [11]. Therefore, we choose the dosage of BTS at 25-fold of the daily dose for humans. All samples were dissolved in H_2_O, and for control group, H_2_O was orally administered instead of BTS extract. One min after sample administration, fructose (Fujifilm) dissolved in H_2_O at a dose of 2.0 g/kg was orally administered. Just before or 0.5, 1, 1.5, 2, and 4 h in rats or 0.5 h in mice after the administration of fructose, animals were anesthetized with inhalation of isoflurane (Viatris, Canonsburg, PA, USA), and blood samples were collected from the jugular vein in rats and the portal vein in mice. Plasma fructose levels were measured using an EnzyChrom Fructose Assay Kit (BioAssay Systems LLC, Hayward, CA, USA).

### HPLC analysis for RR

The standard compounds ( +)-catechin, (–)-epicatechin gallate (ECG), (–)-epigalocatechin gallate (EGCG), (–)-epigalocatechin (EGC), and sennoside A were obtained from Nacalai Tesque (Kyoto, Japan) and were dissolved in ethanol. The extract of RR (5 µg), or the mixture containing ( +)-catechin, ECG, EGCG, EGC, and sennoside A (0.02, 0.1, or 0.2 µg, respectively) were injected into HPLC with the following conditions: column, Cosmosil Cholester (4.5 × 150 mm, Nacalai); mobile phase, 0.5% formic acid in H_2_O/0.5% formic acid in CH_3_CN 90:10 (0 min) – 70:30 (20 min) – 70:30 (22 min), linear gradient; flow rate, 1.0 ml/min; column temperature, 40 °C; and detection, 280 nm. ( +)-catechin, ECG, EGCG, EGC, and sennoside A were eluted at 7.5 min, 11.4 min, 15.3 min, and 19.8 min, respectively. A chromatogram of the extract of RR is shown in Supplementary Fig. 2. Using the linear standard lines for each compound, the concentrations of ( +)-catechin, ECG, EGCG, and sennoside A in the extract of RR were measured.

### Statistical analysis

Data represents mean ± standard error (S.E.). All statistical analyses were performed using Excel Statistical Analysis (version 3.0; Esumi, Tokyo, Japan). Statistical analysis was conducted using Student's *t*-test for the differences between two groups and one-way analysis of variance (ANOVA) followed by Tukey–Kramer’s test for the differences among multiple groups. Difference in kinetics between the two groups were analyzed using a two-way ANOVA. *P*-values less than 0.05 were considered statistically significant.

## Results and discussion

The BTS extract was orally administered to rats at doses equivalent to BTS corresponding to 25-fold the daily dose for humans (2.5 g/kg), and 1 min after the administration, fructose (2.0 g/kg) was orally administered. Blood samples were collected from the jugular vein 0.5, 1, 2, and 4 h after the administration of fructose. Treatment with BTS extract was observed to reduce the absorption of fructose from the intestine (Fig. 1). Two-way ANOVA indicated a significant main effect of BTS treatment (F_1,33_ = 25.4, *p* < 0.001), time (F_5,33_ = 14.8, *p* < 0.001), and interaction between BTS treatment and time (F_5,33_ = 4.58, *p* < 0.001). The area under the concentration (AUC) of fructose from 0 to 4 h was 694 ± 107 µM·hr for the control group (n = 4) and 334 ± 69 µM·hr for the BTS-treated group (*n* = 3), and p < 0.05 was observed between control and BTS-treated group evaluated by Student's *t*-test.

**Fig. 2:**
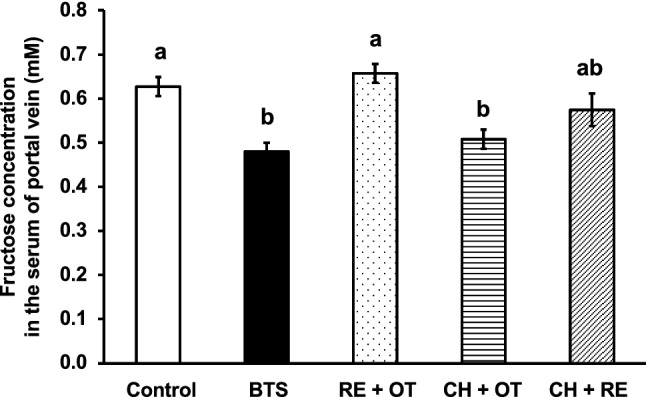
Effect of the extracts of BTS or its component groups on the absorption of fructose in mice. The extracts of BTS (the mixture of CH + RE + OT), the mixtures of CH + OT, RE + OT, and RE + CH, respectively, were prepared, and the samples at the doses equivalent to BTS corresponding to 25-fold of the daily dose for humans (2.5 g/kg) were orally administered to mice. One minute after sample administration, fructose (2.0 g/kg) was orally administered. Twenty-five minutes after fructose administration, mice were anesthetized, and 30 min after fructose administration, blood samples were collected from the portal vein. Abbreviations are presented in Table 1. Each column represents mean ± S.E. (n = 6 for each group). Different alphabetical letters a and b indicate statistically significant differences at *p* < 0.05 between each group evaluated by Tukey–Kramer’s test

Mice were used to determine the inhibitory effect of BTS components on the absorption of fructose, and the blood samples were collected 30 min after the oral administration of fructose. To evaluate the contribution of each crude drug component in BTS, 18 crude drugs were separated into three groups, each containing six crude drugs for clearing heat (CH) group, releasing exterior (RE) group, and others (OT), as shown in Table 1, according to the theory of traditional Chinese medicine (TCM). The "exterior" is the pattern name #SE76 registered in International Classification of Diseases 11th Revision (ICD-11) published from World Health Organization, and is the symptom developed by the cold or heat *evil* present at the surface of body [12]. Although the main pharmacological action of CR is resolving *blood* stasis, CR is classified into RE group in this study because CR also has the effect of removing *wind* and releasing exterior in the theory of TCM [13]. As shown in Fig. 2, BTS extract at 2.5 g/kg significantly reduced the absorption of fructose, and the results obtained using rats were reproduced in mice. Among the BTS component groups, the extract of CH and OT groups significantly reduced the inhibitory effect of the BTS extract on the absorption of fructose, and the effect of the extract of CH and OT was similar to that of the control, suggesting that the active crude drugs in BTS must be present in the CH group.

The traditional effect of CH is to calm or purge the *heat* and *fire*, which are the conditions with grossly visible manifestation of *heat* such as bleeding, flushed face, red eyes, internal clumping, etc. [13]. The approved indications of BTS such as flabby belly, constipation, hyperchlorhydria, arteriosclerosis, hypertension, rush of blood to the head, and obesity are related to the conditions of internal *heat* and *fire*, and the effect of CH is considered to be the main effect of BTS. The preventive effect of BTS on the absorption of fructose is related to the prevention of obesity, it is reasonable that crude drugs in CH group exhibited this effect in the theory of traditional medicine.

**Fig. 3:**
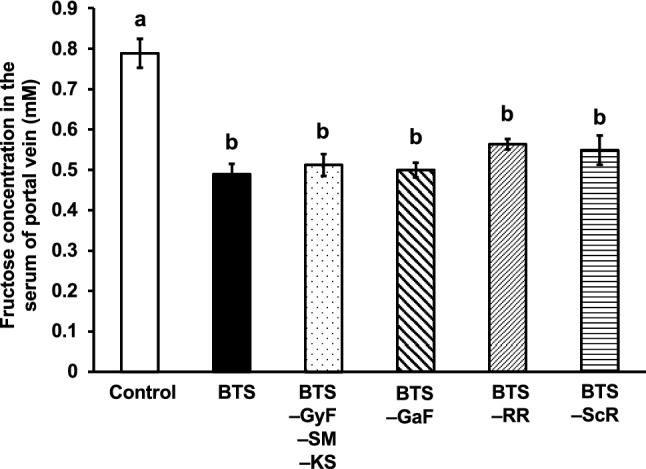
Effect of the extracts of BTS and the mixtures of each crude drug in CH on the absorption of fructose in mice. The extracts of each crude drug mixture were prepared, and doses equivalent to 25-fold of the daily dose of BTS for humans (2.5 g/kg), were orally administered to mice. One minute after sample administration, fructose (2.0 g/kg) was orally administered. Twenty-five minutes after fructose administration, mice were anesthetized, and 30 min after fructose administration, blood samples were collected from the portal vein. Abbreviations are presented in Table 1. Each column represents mean ± S.E. (*n* = 6 for each group). Different alphabetical letters a and b indicate statistically significant differences at *p* < 0.05 between each group evaluated by Tukey–Kramer’s test

The CH group included RR, GaF, ScR, GyF, SM, and KS. As GyF, SM, and KS are crude drugs derived from minerals, we created a sub-group of mineral crude drugs in CH. To determine the contribution of each crude drug or subgroup to the effect of the CH group, we prepared the extract of BTS without RR, GaF, ScR, or the sub-group of CH. As shown in Fig. 3, the extract of BTS without RR slightly recovered the inhibitory effect of the BTS extract, suggesting that RR might contribute the effect of BTS; however, the removal of each RR, GaF, ScR, or the sub-group of CH from BTS did not exhibit any significant differences from the effect of BTS. It has been suggested that the combination and the interaction among RR, GaF, ScR, and the sub-group of mineral crude drugs play important roles in the inhibitory effect of BTS on fructose absorption.

**Fig. 4:**
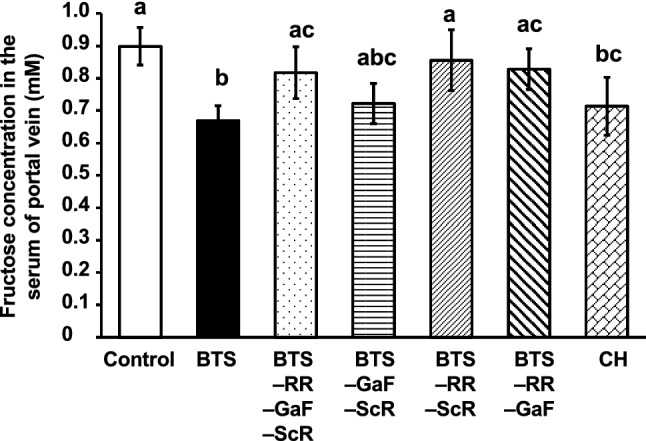
Effect of the extracts of BTS and the mixture of each crude drug in CH group on the absorption of fructose in mice. The extracts of each crude drug mixture were prepared, and the samples at doses equivalent to 25-fold of the daily dose of BTS for humans (2.5 g/kg), were orally administered to mice. One minute after sample administration, fructose (2.0 g/kg) was orally administered. Twenty-five minutes after fructose administration, mice were anesthetized, and 30 min after fructose administration, blood samples were collected from the portal vein. A abbreviations are presented in Table 1. Each column represents mean ± S.E. (*n* = 6 for each group). Different alphabetical letters a and b indicate statistically significant differences at *p* < 0.05 between each group evaluated by Tukey–Kramer’s test

In the next experiment, we focused on RR, GaF, and ScR as crude drugs derived from plants in the CH group and prepared the extract of BTS without these three crude drugs, or three pairs of these crude drugs. We also prepared an extract of six crude drugs from the CH group. We evaluated their inhibitory effects on the absorption of fructose. As shown in Fig. 4, the extract of BTS without these three drugs, the pair of RR and ScR, and the pair of RR and GaF exhibited a significantly weaker inhibitory effect on fructose absorption compared to the extract of BTS. CH extract significantly inhibited fructose absorption. These results highlight the importance of RR in BTS and suggest that RR significantly contributed the inhibitory effect of BTS on fructose absorption.

**Fig. 5:**
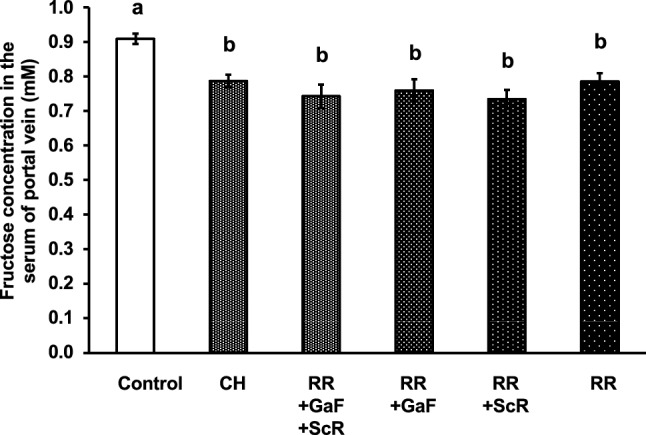
Effect of the extracts of crude drug mixture in CH group with Rhei Rhizoma (RR) on the absorption of fructose in mice. The extracts of each crude drug mixture were prepared, and the samples at the doses equivalent 25-fold of the daily dose of BTS for humans (2.5 g/kg), were orally administered to mice. One minute after sample administration, fructose (2.0 g/kg) was orally administered. Twenty-five minutes after fructose administration, mice were anesthetized, and 30 min after fructose administration, blood samples were collected from the portal vein. Abbreviations are presented in Table 1. Each column represents the mean ± S.E. (*n *= 6 for each group). Different alphabetical letters a and b indicate statistically significant differences at *p* < 0.05 between each group evaluated by Tukey–Kramer’s test

Next, we prepared an extract of single RR, the pairs of RR and GaF, RR and ScR, or the combination of RR, GaF, and ScR, and evaluated their inhibitory effects on fructose absorption. As shown in Fig. 5, all extracts containing RR exhibited significant inhibitory effects on fructose absorption, and no significant interactions between RR and other crude drugs were observed. It was revealed that a single RR mainly contributes to the inhibitory effect of BTS on fructose absorption. However, since the removal of RR from BTS did not significantly reduce the inhibitory effect of BTS, it is predicted that the combination of some crude drugs in RE and OT would interact with RR, and that this interaction would partially contribute to the inhibitory effect of BTS on fructose absorption.

**Fig. 6:**
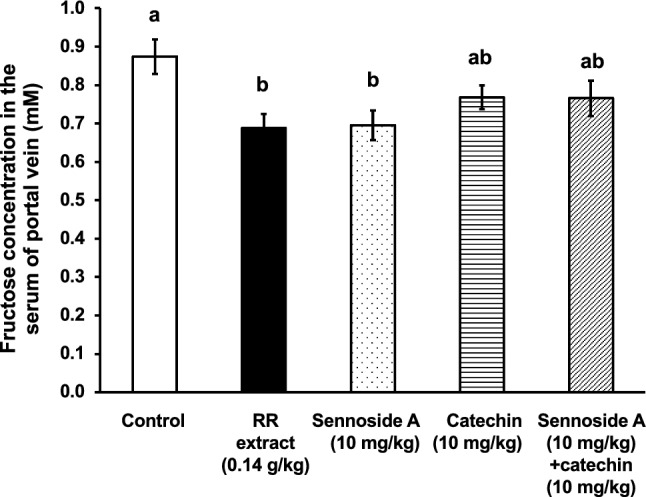
Effect of the extracts of Rhei Rhizoma (RR) and its constituents on the absorption of fructose in mice. The extract of RR at the doses (0.14 g/kg) equivalent to 25-fold of the daily dose of BTS for humans (2.5 g/kg), sennoside A and/or ( +)-catechin (0.01 g/kg) were orally administered to mice. One minute after sample administration, fructose (2.0 g/kg) was orally administered. Twenty-five minutes after fructose administration, mice were anesthetized, and 30 min after fructose administration, blood samples were collected from the portal vein. Each column represents the mean ± S.E. (*n* = 6 for each group). Different alphabetical letters a and b indicate statistically significant differences at *p* < 0.05 between each group evaluated by Tukey–Kramer’s test

Among the constituents of RR, we focused on ( +)-catechin and sennoside A based on their content in the extract because these compounds are two major representatives of catechins including ECG, EGC, and EGCG and anthraquinones including emodin and chrysophanol. As the contents of ( +)-catechin, ECG, EGCG, and sennoside A in the present RR extract were 1.0, 0.21, 0.48, and 1.6% (w/w), respectively, and EGC was not detected. The extract of RR at the doses (0.14 g/kg), which corresponds to 25-fold of the daily dose for humans (2.5 g/kg), is equivalent to the dosages of ( +)-catechin (1.4 mg/kg) and sennoside A (2.2 mg/kg). However, because we were cautious that the activities of each ingredient might be dispersed and that the significant activities of a single ingredient might not appear, we adopted the dosages of ( +)-catechin and sennoside A of 10 mg/kg. At these dosages, sennoside A significantly inhibited the absorption of fructose, whereas ( +)-catechin did not. In contrast, the combination of ( +)-catechin and sennoside A did not exhibit a significant effect (Fig. 6). Since there was no statistical significance between the group of sennoside A and the group of sennoside A + ( +)-catechin, it can be said that ( +)-catechin did not exhibit any effects on the inhibitory effect of sennoside A. However, the significant inhibitory effect of sennoside A was lost by the combination with ( +)-catechin. It is considered that ( +)-catechin may counteract the effect of sennoside A by some unknown mechanisms, and further studies to clarify the interaction between sennoside A and ( +)-catechin are needed.

**Fig. 7:**
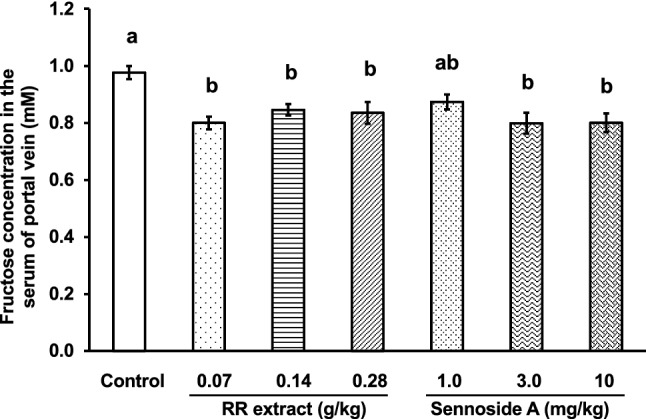
Effect of the extract of Rhei Rhizoma (RR) and sennoside A on the absorption of fructose in mice. Extracts of RR or sennoside A were orally administered to the mice. One minute after sample administration, fructose (2.0 g/kg) was orally administered. Twenty-five minutes after fructose administration, mice were anesthetized, and 30 min after fructose administration, blood samples were collected from the portal vein. Each column represents the mean ± S.E. (*n* = 6 for each group). Different alphabetical letters a and b indicate statistically significant differences at *p* < 0.05 between each group evaluated by Tukey–Kramer’s test

We evaluated the dose-dependency of RR extract and sennoside A. As shown in Fig. 7, the RR extract exhibited a significant inhibitory effect on absorption; however, dose-dependency did not appear, and the effect was saturated. Sennoside A significantly inhibited the absorption of fructose in a dose-dependent manner, and the effect was almost saturated at a dose of 3 mg/kg. Based on the content of sennoside A in the RR extract, the dosage of the 0.07 g/kg RR extract was equivalent to 1.1 mg/kg sennoside A and 0.7 mg/kg ( +)-catechin. Although the differences between the inhibitory effects of RR extract (0.07 g/kg) and that of sennoside A (1.0 mg/kg) were not statistically significant, sennoside A would not wholly contribute to the effect of RR extract and there would be other active ingredients than sennoside A in RR extract because the activities of RR extract was saturated at this dosage. Furthermore, the counteractive effect of ( +)-catechin on the effect of sennoside A and other active ingredients would not be appeared at this small dosage. However, because the differences of the activities from that of control were low, we consider that statistically significant results would hardly be obtained at low dosages (< 0.07 g/kg and 1 mg/kg) of RR extract and sennoside A, respectively. We conclude that sennoside A partially contributes to the inhibitory effect of RR, however, the contribution of other ingredients may also be possible, and remains to be explored.

In a previous in vitro study [9], we revealed that the extract of BTS significantly inhibited GLUT5, which transports fructose from the intestinal lumen into epithelial cells and plays a role in the absorption of fructose in the intestine. The present in vivo study reproduced these in vitro results. However, although the extract of RR exhibited a significant inhibitory effect of GLUT5 in a previous in vitro study, the effect was not strong, and other crude drug extracts of ZR, SaR, PlR, MH, GaF, and CR exhibited stronger effects than the RR extract [9]. The mechanisms of fructose absorption may not be fully explained by the function of GLUT5 in vivo, and mechanisms other than GLUT5 could contribute to the absorption of fructose.

In conclusion, BTS extract significantly inhibited the absorption of fructose in rats and mice, and among its crude drug the components, RR mainly contributes to this activity of BTS and some crude drugs except for the drugs for clearing *heat* group in BTS partially support this activity. Sennoside A partially contributes the activity of RR, and other unknown ingredients may also support the activity of RR. These results may explain one of the action mechanisms of BTS when used for the treatment of obesity in clinics and drug stores.

## Data Availability

The data used to support the findings of this study are available from the corresponding author upon request.
